# White Matter Abnormalities of Auditory Neural Pathway in Sudden Sensorineural Hearing Loss Using Diffusion Spectrum Imaging: Different Findings From Tinnitus

**DOI:** 10.3389/fnins.2020.00200

**Published:** 2020-03-25

**Authors:** Zihao Zhang, Xiuqin Jia, Xiaojiao Guan, Yi Zhang, Yuelei Lyu, Jing Yang, Tao Jiang

**Affiliations:** ^1^Department of Radiology, Beijing Chaoyang Hospital, Capital Medical University, Beijing, China; ^2^Department of Radiology, Beijing Shijitan Hospital, Capital Medical University, Beijing, China; ^3^Department of Hyperbaric Oxygen, Beijing Chaoyang Hospital, Capital Medical University, Beijing, China

**Keywords:** diffusion spectrum imaging, tractography, sudden sensorineural hearing loss, demyelination, white matter

## Abstract

Sudden sensorineural hearing loss (SSNHL) is a complex and challenging emergency which requires evidence regarding its pathophysiological changes to guide the treatment. The aim of this study was to evaluate the white matter integrity of the auditory neural pathway in patients with unilateral SSNHL in acute stage by using diffusion spectrum imaging tractography. In the present study, 60 individuals with acute SSNHL (29 males, 50.7 ± 11.8 years) and 25 healthy controls (13 males, 45.2 ± 13.2 years) underwent diffusion spectrum imaging tractography and high resolution T1 structural examinations using a 3T magnetic resonance imaging system. The areas of the auditory neural pathway were defined as regions of interest (ROIs). The quantitative anisotropy (QA) and the generalized fractional anisotropy (GFA) were compared between the patients with unilateral SSNHL and controls in these ROIs. We further evaluated the correlation between the parameter values and hearing loss level. The mean pure tone audiometry of patients at the onset presentation was 63.2 ± 26.2 dB. The right-sided SSNHL was involved in 25 (41.7%) cases and the left-sided in 35 (58.3%) cases. The QA values in the contralateral medial geniculate body, the bilateral anterior corona radiata and the anterior limb of internal capsule were significantly reduced in SSNHL patients compared to controls. In addition, the decrease QA value of the contralateral medial geniculate body was related to the increase severity of disease, even after controlling potential confounding factors. The present study demonstrated that patients with SSNHL exhibited altered integrity of white matter in the auditory neural pathway. Furthermore, the decreased QA values in the contralateral medial geniculate body might predict the severity of this disease. In the present study, tinnitus has not been found to effect in brain area obviously.

## Introduction

Sudden sensorineural hearing loss (SSNHL) is an emergency of otology. It is defined as hearing loss of at least 20 dB in two contiguous frequencies within 72 h (Editorial Committee of Chinese Journal of Otorhinolaryngology Head and Neck Surgery, and Chinese Academy of Otorhinolaryngology Head and Neck Surgery Branch, 2015). SSNHL is no longer a rare disease. Some studies have reported an annual incidence of 160 cases per 100,000 (Klemm et al., 2009). So far, the pathogenesis of the disease is unclear, and some theories have been put forward to explain this clinical problem, including the causes of infection, vascular occlusion, immune-mediated mechanisms, coagulation disorders, and breaks of labyrinthine membranes (Lin et al., 2015; Ajduk et al., 2017). The unknown etiologies lead to many empirical treatment options. Therefore, the pathophysiology of sudden hearing loss is of great significance for early targeted treatment of patients (Atay et al., 2016). However, the cause of SSNHL can be identified by conventional MR only in the presence of severe interruption of the interaxial auditory pathway, such as tumors and infarcts. In the absence of such lesions, it is difficult to determine the cause of SSNHL in conventional MR.

Conventional diffusion tensor imaging (DTI) is a sensitive and non-invasive method to evaluate white matter fiber. It can reconstruct fiber pathways to assessing the abnormalities in the central nervous system. In the past, some researches have already used DTI to study the neural changes in patients with sensorineural hearing loss (Lin et al., 2008; Wu et al., 2009; Huang et al., 2015; Tarabichi et al., 2017). But DTI cannot resolve the beginning and end point of nerve fiber or the fiber crossings in the white matter, resulting in artifacts and wrong tracts (Fernandez-Miranda et al., 2012). Diffusion spectrum imaging (DSI) (Wedeen et al., 2005; Hagmann et al., 2007; Schmahmann et al., 2007) is a modeless technique. It uses ODF (orientation distribution function) to consider the diffusion patterns in each voxel, which has been shown to better resolve the ambiguity of fiber crossing encountered in tractography. In DSI, diffusion weighted images are organized in the q-space (integral points of a cubic lattice) in the sphere and can be obtained in different directions. It proposes the probability density function (PDF) to describe the diffusion in each voxel, which specifies the three-dimensional distribution of microscopic displacements of water molecules (Wedeen et al., 2005). Through GQI (generalized q-sampling imaging) reconstruction, we can obtain the parameter of ODF, including quantitative anisotropy (QA) and generalized factional anisotropy (GFA). QA is defined as the number of anisotropic spins diffused along the fiber direction which can identify the fault structure well of cross fiber (Yeh et al., 2010). GFA reflecting the integrity of the neural fiber bundles and higher value indicates that more consistent diffusion direction of water molecules along fiber bundle (Tuch, 2004; Lo et al., 2011). Recently DSI is more and more used to study the brain nerve structure (Lin et al., 2014; Panesar et al., 2017; Yue et al., 2017). The aim of this study was to investigate the changes in the auditory neural pathway in patients with acute SSNHL by DSI tractography and measuring these DSI parameters.

## Materials and Methods

### Patients

This prospective, clinical trial involved 25 healthy controls (13 males, 45.2 ± 13.2 years) and 60 patients (29 males, 50.7 ± 11.8 years) diagnosed with unilateral idiopathic SSNHL of Beijing Chaoyang Hospital, between November 2016 and July 2009. The criteria of SSNHL were the presence of a sensorineural hearing loss greater than or equal to 20 dB or exceed at least two adjacent audiometric frequencies, which developed within a few hours but no more than 2 weeks. The uniform radiological method was magnetic resonance imaging, but temporal computed tomography had also been obtained from some patients. Based on radiologic diagnosis combined with clinical information, all patients were diagnosed by clinicians. Exclusion criteria were as follows: (1) patients with abnormal anatomy, (2) otitis media, cholesteatoma and other middle ear diseases, or severe cerebral infarction and other neuromuscular or systemic diseases, (3) otological surgery history, (4) ototoxic medication history. The study was approved by the ethics committee of Beijing Chaoyang Hospital, Capital Medical University. Informed consent for the participation to the study was received from all patients.

### Clinical Characteristics

The demographic data, smoking status, associated symptoms and comorbidities were recorded. The pure tone audiometry (interlacoustics; amplifon) was used to evaluate the hearing level in the sound insulation room. Hearing loss is defined as pure tone average (PTA), which follows the standards of the World Health Organization (WHO-1997).

### Image Acquisition and Reconstruction

DSI data were acquired on 3T Prisma (Siemens) using a 64-channel coil. This involved a 15 min, 257-direction scan using a twice-refocused spin-echo EPI sequence and multiple q values (Wedeen et al., 2005) [echo time (TE) = 79 ms, repetition time (TR) = 7200 ms, voxel size = 2.2 × 2.2 × 2.2 mm, field of view = 220 × 220 mm, bmax = 3000 s/mm^2^]. We also used high-resolution anatomical imaging as an anatomical comparison, using a 9-min T1-weighted magnetization-prepared rapid gradient echo (TE = 2.27 ms, TR = 2300 ms, 208 slices, flip angle = 8, field of view = 256 × 256 mm^2^, voxel size = 1.0 × 1.0 × 1.0 mm^3^). A generalized Q-sampling imaging approach (Yeh et al., 2010) were used to reconstructed DSI data. The orientation distribution functions were reconstructed to 362 discrete sampling directions with an average diffusion distance of 1.2 mm. CSF calibration uses spatial normalization to identify the position of CSF and use it as a free water diffusion to unify the amount of diffusion relative to it.

### Fiber Tracking and Analysis

DSI Studio (an open-source diffusion MRI analysis tool available for free download at http://dsi-studio.labsolver.org) was used for fiber tracking. A whole brain seeding approach using multiple region of interest (ROI) masks was performed. In voxels with multiple fiber orientations, fiber tracking was initiated separately for each orientation, and fiber progression continued with a step size of 1.0 mm, minimum fiber length of 20 mm and turning angle threshold of 60. If multiple fiber orientations existed in the current progression location, the fiber orientation that was nearest to the incoming direction and formed a turning angle smaller than 60 was selected to determine the next moving direction (Fernandez-Miranda et al., 2012). To smooth each track, the next moving directional estimate of each voxel was weighted by 20% of the previous incoming direction and 80% of the nearest fiber orientation. This progression was repeated until the quantitative anisotropy of the fiber orientation dropped below a preset threshold (0.13–0.17 depending on the subject) or there was no fiber selected within the 60 angular range in the progression (Yeh et al., 2013). The ROI selected in the auditory neural pathway was shown in Table 1. The T1 and DSI images were spatially normalized into MNI. Then, an optical fiber tracking algorithm based on streamline is implemented by using DSI studio software, and the QA and GFA parameters of each ROI were obtained.

**TABLE 1:** The selected ROI and how they were defined.

ROI	Template	MNI coordinates (x, y, z)	Volume (bilateral, cm^3^)
Superior olivary nucleus	–	±13, −35, −41	5
Inferior colliculus	–	±6, −33, −11	5
Medial geniculate bodies	–	±17, −24, −2	8
Heschl	AAL	–	–
Superior temporal gyrus	AAL	–	–
Meddle temporal gyrus	AAL	–	–
Inferior tempotal gyrus	AAL	–	–
Anterior limb of internal capsule	JHU-WhiteMatter-Labels	–	–
Posterior limb of internal capsule	JHU-WhiteMatter-Labels	–	–
Anterior corona radiata	JHU-WhiteMatter-Labels	–	–
Posterior corona radiata	JHU-WhiteMatter-Labels	–	–
Lateral lemniscus	HCP842-tractography	–	–
Acoustic radiation	HCP842-tractography	–	–
Brodmann 41	Brodmann	–	–
Brodmann 42	Brodmann	–	–

### Statistical Analysis

Continuous variables were summarized as means (± standard deviation). For categorical variables, the percentages of patients in each category were calculated.

Comparison of DSI parameters between patients and control group, with the usage of Student’s *t*-test, as appropriate was to make the comparisons between subgroups of left-sided and right-sided. Results were corrected for multiple comparisons using the Bonferroni correction with a significance level of *p* < 0.05/N, where N was the number of ROIs.

Bivariate correlation and partial correlation analyses were used to identify the relationship between the DSI parameters of ROIs that exhibited significant differences and clinical severity assessment. A *P* < 0.05 was considered statistically significant. All data were analyzed by SPSS software version 19.0 of Windows (SPSS Inc. Chicago, United States).

## Results

Table 2 showed the patients’ demographic details, clinical conditions and hearing test results at admission. In total, there were 60 patients included 29 males and 31 females, with a mean age of 50.7 ± 11.8 years. The right-sided SSNHL was involved in 25 (41.7%) cases and the left-sided in 35 (58.3%) cases. And 52 (86.7%) exhibited tinnitus, 21 (35.0%) exhibited dizzy as accompanying symptoms. Of total, 28 (47.5%) patients had cerebral ischemia and 21 (35.0%) had hypertension, which is the most common Comorbidities. Mean pure tone audiometry at the onset presentation was 63.2 ± 26.2 dB, of which 26.7% was mild, 11.7% was moderate, 13.3% was moderate-severe, 28.3% was severe and 20.0% was extremely severe. The audiogram type was low-frequency in 16.7%, high-frequency in 26.7%, and all-frequency in 55.0% cases.

**TABLE 2:** Characteristics of patients with unilateral sudden sensorineural hearing loss.

Clinical characteristic	Total	Left	Right
**Age, Years**			
Mean (SD)^†^	50.7 ± 11.8	50.1 ± 11.3	51.5 ± 12.7
Male sex, No. (%)	29 (48.3)	15 (42.9)	14 (56.0)
**Side, NO. (%)**			
Right	25 (41.7)	—	—
Left	35 (58.3)	—	—
Ever smoking, No. (%)	13 (21.7)	4 (11.4)	9 (36.0)
**Length of hospital admission**			
Mean (SD)	8.8 ± 4.3	8.9 ± 4.2	8.6 ± 4.5
**Accompanying symptom, NO. (%)**			
Tinnitus	52 (86.7)	30 (85.7)	22 (88.0)
Dizzy	21 (35.0)	16 (45.7)	5 (20.0)
**Comorbidities, NO. (%)**			
Cerebral ischemia	28 (47.5)	18 (51.4)	10 (41.7)
Hypertension	21 (35.0)	11 (31.4)	10 (40.0)
Type 1 or 2 diabetes	10 (16.7)	8 (22.9)	2 (8.0)
Hyperlipidemia	16 (26.7)	12 (34.3)	4 (16.0)
**Severity assessment, NO. (%)**			
Mild	16 (26.7)	8 (22.9)	8 (32.0)
Moderate	7 (11.7)	4 (11.4)	3 (12.0)
Moderate-severe	8 (13.3)	5 (14.3)	3 (12.0)
Severe	17 (28.3)	9 (25.7)	8 (32.0)
Extremely severe	12 (20.0)	9 (25.7)	3 (12.0)
**Type**			
Low-frequency hearing loss	10 (16.7)	7 (20.0)	3 (12.0)
High-frequency hearing loss	16 (26.7)	9 (25.7)	7 (28.0)
All-frequency hearing loss	33 (55.0)	19 (54.3)	14 (56.0)
**Initial pta Score^‡^**			
Mean (SD)	63.2 ± 26.2	66.1 ± 26.6	59.1 ± 25.7
Relapse, No. (%)	6 (10.0)	5 (14.3)	1 (4.0)

*^†^Plus–minus values are means ± SD, ^‡^PTA means pure tone audiometry.*

**FIGURE 1:**
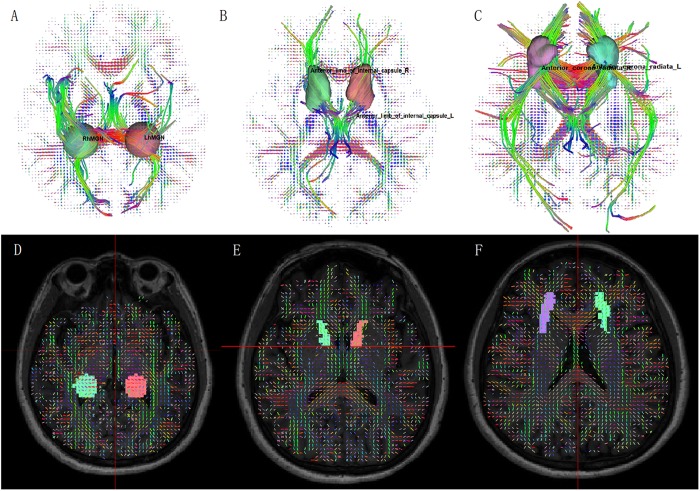
Fiber tractography from diffusion spectrum imaging: **(A)** medial geniculate body; **(B)** anterior limb of internal capsule; **(C)** anterior corona radiata. ROI on QA map: **(D)** medial geniculate body; **(E)** anterior limb of internal capsule; **(F)** anterior corona radiata.

DSI tractography measures of QA and GFA for each ROI of patients with SSNHL and controls were shown in Tables 3, 4. Compared with healthy group, the QA values of the contralateral (i.e., opposite to the lesion side) medial geniculate body, the bilateral anterior corona radiata and anterior limb of internal capsule were obviously reduced (*P* < 0.002) both for the left-sided and the right-sided SSNHL group (Figure 1). However, the GFA values of the bilateral anterior corona radiata and anterior limb of internal capsule decreased significantly only in the right-sided group (*P* < 0.002). No significant differences were identified between the left-sided SSNHL and controls. The QA and GFA values were compared between patients with or without tinnitus and dizziness by independent sample *t*-test, and found there was no difference in these areas (*p* > 0.05), as shown in the Supplementary Table S1.

**TABLE 3:** The QA^†^ values obtained from the left and right sides of the subjects with SSNHL^‡^ and of the normal group.

Variable	Normal *n* = 25	Left-sided SSNHL *n* = 35	*P*^§^	Right-sided SSNHL *n* = 25	*P*^§^
**Superior olivary nucleus**					
Left	0.6726 ± 0.0784	0.5857 ± 0.1228	0.003	0.6597 ± 0.0889	0.589
Right	0.6584 ± 0.0910	0.5957 ± 0.1142	0.027	0.6209 ± 0.1027	0.178
**Inferior colliculus**					
Left	0.6531 ± 0.0800	0.6127 ± 0.1	0.100	0.6374 ± 0.0942	0.527
Right	0.6463 ± 0.0828	0.5897 ± 0.0994	0.024	0.6210 ± 0.0966	0.325
**Medial geniculate bodies**					
Left	0.6324 ± 0.0815	0.5721 ± 0.1042	0.019	0.5497 ± 0.0984	0.002*
Right	0.6328 ± 0.0924	0.5487 ± 0.0873	0.001*	0.5679 ± 0.1090	0.028
**Lateral lemniscus**					
Left	0.6531 ± 0.0816	0.6159 ± 0.0985	0.127	0.6103 ± 0.0888	0.082
Right	0.6486 ± 0.0688	0.5733 ± 0.1131	0.002*	0.6019 ± 0.0881	0.042
**Anterior limb of internal capsule**					
Left	0.5718 ± 0.0755	0.4957 ± 0.0741	*P* < 0.001*	0.4762 ± 0.0969	P < 0.001*
Right	0.5872 ± 0.0687	0.5101 ± 0.076	*P* < 0.001*	0.4934 ± 0.0941	P < 0.001*
**Posterior limb of internal capsule**					
Left	0.6947 ± 0.0635	0.6546 ± 0.1225	0.105	0.6501 ± 0.1007	0.067
Right	0.6907 ± 0.0551	0.6619 ± 0.1147	0.202	0.6580 ± 0.1175	0.213
**Heschl**					
Left	0.6055 ± 0.0917	0.5472 ± 0.1012	0.026	0.5492 ± 0.0822	0.027
Right	0.5793 ± 0.0764	0.4771 ± 0.1037	*P* < 0.001*	0.4813 ± 0.1449	0.004
**Superior temporal gyrus**					
Left	0.5975 ± 0.0762	0.5408 ± 0.0876	0.012	0.5337 ± 0.0851	0.008
Right	0.5942 ± 0.0732	0.5457 ± 0.087	0.027	0.5299 ± 0.0753	0.004
**Meddle temporal gyrus**					
Left	0.6021 ± 0.0752	0.5575 ± 0.0769	0.029	0.5421 ± 0.0764	0.007
Right	0.6065 ± 0.0721	0.5509 ± 0.0815	0.008	0.5504 ± 0.0794	0.012
**Inferior temporal gyrus**					
Left	0.5595 ± 0.0661	0.5104 ± 0.069	0.008	0.4937 ± 0.0778	0.002*
Right	0.5732 ± 0.0687	0.4997 ± 0.1027	0.003	0.5070 ± 0.0749	0.002*
**Anterior corona radiata**					
Left	0.5869 ± 0.0718	0.5218 ± 0.0743	0.001*	0.4967 ± 0.1073	0.001*
Right	0.5712 ± 0.0638	0.5154 ± 0.0661	0.002*	0.5069 ± 0.0633	0.001*
**Posterior corona radiata**					
Left	0.7173 ± 0.0815	0.6794 ± 0.11	0.150	0.6706 ± 0.1078	0.090
Right	0.7081 ± 0.0785	0.6668 ± 0.103	0.084	0.6660 ± 0.1086	0.124
**Acoustic radiation**					
Left	0.6728 ± 0.0784	0.6226 ± 0.0928	0.032	0.6156 ± 0.0916	0.022
Right	0.6617 ± 0.0827	0.6093 ± 0.0909	0.026	0.5945 ± 0.0942	0.010
Brodman 41 area	0.6350 ± 0.0790	0.5662 ± 0.1208	0.016	0.5720 ± 0.0766	0.006
Brodman 42 area	0.5483 ± 0.0793	0.4885 ± 0.0748	0.004	0.4877 ± 0.0782	0.009

*^†^QA means quantitative anisotropy. ^‡^SSNHL means sudden sensorineural hearing loss.****^§^***The left and right side of SSNHL patients was compared with normal group separately. *The asterisk denotes a significant effect under the conservative Bonferroni correction controlling the family-wise error rate among all the morphology effects hypothesis tests at 5% (adjusted threshold p = 0.05/28∼0.002.

**TABLE 4:** The GFA^†^ values obtained from the left and right sides of the subjects with SSNHL^‡^ and of the normal group.

Variable	Normal *n* = 25	Left-sided SSNHL *n* = 35	*P*^§^	Right-sided SSNHL *n* = 25	*P*^§^
**Superior olivary nucleus**					
Left	0.0916 ± 0.0068	0.0876 ± 0.0157	0.187	0.0986 ± 0.0085	0.002*
Right	0.0906 ± 0.0077	0.0885 ± 0.0171	0.581	0.0943 ± 0.0072	0.085
**Inferior colliculus**					
Left	0.0941 ± 0.0055	0.0936 ± 0.0121	0.835	0.0982 ± 0.0046	0.007
Right	0.0941 ± 0.0054	0.0942 ± 0.0067	0.928	0.0966 ± 0.0060	0.132
**Medial geniculate bodies**					
Left	0.1002 ± 0.0096	0.0995 ± 0.0112	0.802	0.0944 ± 0.0109	0.048
Right	0.0976 ± 0.0080	0.0950 ± 0.0098	0.296	0.0950 ± 0.0111	0.355
**Lateral lemniscus**					
Left	0.0936 ± 0.0084	0.0945 ± 0.0067	0.653	0.0949 ± 0.0106	0.649
Right	0.0917 ± 0.0062	0.0906 ± 0.0090	0.589	0.0939 ± 0.0155	0.522
**Anterior limb of internal capsule**					
Left	0.0860 ± 0.0054	0.0825 ± 0.0071	0.046	0.0796 ± 0.0073	0.001*
Right	0.0883 ± 0.0042	0.0841 ± 0.0061	0.004	0.0826 ± 0.0069	0.001*
**Posterior limb of internal capsule**					
Left	0.1050 ± 0.0043	0.0996 ± 0.0068	P < 0.001*	0.1013 ± 0.0043	0.004
Right	0.1048 ± 0.0039	0.1006 ± 0.0054	0.001*	0.1015 ± 0.0075	0.062
**Heschl**					
Left	0.0898 ± 0.0103	0.0891 ± 0.0125	0.805	0.0895 ± 0.0079	0.898
Right	0.0829 ± 0.0147	0.0788 ± 0.0148	0.295	0.0804 ± 0.0221	0.637
**Superior temporal gyrus**					
Left	0.0938 ± 0.0045	0.0903 ± 0.0076	0.030	0.0899 ± 0.0085	0.050
Right	0.0929 ± 0.0069	0.0899 ± 0.0084	0.152	0.0888 ± 0.0063	0.032
**Meddle temporal gyrus**					
Left	0.0947 ± 0.0045	0.0935 ± 0.0061	0.412	0.0924 ± 0.0052	0.098
Right	0.0941 ± 0.0049	0.0907 ± 0.0068	0.039	0.0917 ± 0.0050	0.098
**Inferior temporal gyrus**					
Left	0.0864 ± 0.0094	0.0859 ± 0.0065	0.785	0.0857 ± 0.0080	0.779
Right	0.0890 ± 0.0070	0.0856 ± 0.0066	0.067	0.0859 ± 0.0093	0.199
**Anterior corona radiata**					
Left	0.0955 ± 0.0060	0.0908 ± 0.0076	0.012	0.0892 ± 0.0076	0.002*
Right	0.0940 ± 0.0048	0.0895 ± 0.0068	0.006	0.0886 ± 0.0064	0.001*
**Posterior corona radiata**					
Left	0.1127 ± 0.0095	0.1094 ± 0.0081	0.155	0.1106 ± 0.0098	0.456
Right	0.1120 ± 0.0104	0.1079 ± 0.0085	0.102	0.1090 ± 0.0113	0.348
**Acoustic radiation**					
Left	0.1033 ± 0.0071	0.1019 ± 0.0080	0.483	0.1011 ± 0.0092	0.345
Right	0.1017 ± 0.0077	0.0996 ± 0.0089	0.329	0.0995 ± 0.0095	0.363
Brodman 41 area	0.0969 ± 0.0040	0.0959 ± 0.0070	0.543	0.0958 ± 0.0070	0.534
Brodman 42 area	0.0845 ± 0.0088	0.0813 ± 0.0080	0.151	0.0811 ± 0.0078	0.153

*^†^GFA means generalized fractional anisotropy. ^‡^SSNHL means sudden sensorineural hearing loss.****^§^***The left and right side of SSNHL patients was compared with normal group separately. *The asterisk denotes a significant effect under the conservative Bonferroni correction controlling the family-wise error rate among all the morphology effects hypothesis tests at 5% (adjusted threshold p = 0.05/28~0.002.

We also further evaluated the correlation between QA values of these three brain regions and clinical severity assessment. Table 5 showed the results of bivariate correlation analysis and partial correlation analysis. We found that the QA value of the contralateral medial geniculate body and the bilateral anterior limb of internal capsule were negatively correlated with the severity of hearing loss (*P* < 0.05). The partial correlation analysis, with controlling including age, gender, smoking, cerebral ischemia, hypertension, diabetes and hyperlipidemia, showed these relationships persisted in the contralateral medial geniculate body (*r* = −0.416, *P* = 0.028 for the left-sided group; *r* = −0.675, *P* = 0.002 for the right-sided group). The results of partial correlation analysis eliminated the influence of these possible risk factors.

**TABLE 5:** Results of correlation analysis between QA^†^ values of selected brain areas and severity assessment of patients with sudden sensorineural hearing loss.

Variable	Left-sided SSNHL^‡^				Right-sided SSNHL			
		
	Bivariate correlation analysis	*P*	Partial correlation analysis*	*P*	Bivariate correlation analysis	*P*	Partial correlation analysis*	*P*
**Medial geniculate bodies**								
Left	–	–	–	–	−0.685	0	−0.675	0.002
Right	−0.447	0.007	−0.416	0.028	–	–	–	–
**Anterior limb of internal capsule**								
Left	−0.358	0.035	−0.135	0.494	−0.411	0.041	−0.380	0.120
Right	−0.350	0.039	−0.138	0.485	−0.345	0.092	−0.249	0.319
**Anterior corona radiata**								
Left	−0.290	0.090	−0.077	0.699	−0.250	0.228	−0.329	0.183
Right	−0.304	0.076	−0.088	0.656	−0.274	0.184	−0.243	0.332

^†^QA means quantitative anisotropy. ^‡^SSNHL means sudden sensorineural hearing loss. *Partial correlation analysis controls age, gender, smoking, cerebral ischemia, hypertension, diabetes and hyperlipidemia.

## Discussion

To the best of our knowledge, the current study firstly applied a high angular resolution diffusion tractography technique of DSI to examine the changes of nerve fibers in the auditory neural pathway in patients with SSNHL. A major finding of this study was that compared with healthy controls, the QA values of the contralateral medial geniculate body, the bilateral anterior corona radiata and anterior limb of internal capsule in patients with sudden deafness were obviously reduced, notably in the contralateral medial geniculate body. In addition, the decrease of QA value of medial geniculate body was significantly related to the increase of disease severity, even after controlling other potential confounding factors. At present, tinnitus and dizziness have not been found to affect these areas on the auditory neural pathway.

The central auditory pathway starts from the cochlear branch of the eighth brain nerve. These synapses firstly enter the brain at the junction of pons and medulla oblongata, and transmit the encoded auditory nerve information to the cochlear nucleus of medulla oblongata. Most of the fibers project to the superior olivary nucleus on the opposite side, and a few of them project to the ipsilateral side. Then the fibers continue to go up through the lateral lemniscus. The efferent fibers project from the inferior colliculus to the medial geniculate body. Finally, the acoustic radiation from the medial geniculate body transmits the auditory information to the temporal cortex through the inner capsule (Wakana et al., 2004; Hernández-Zamora and Poblano, 2014).

The decrease of QA suggests the presence of a demyelinating process in the early stages of disease, and provides imaging verification for the pathophysiological process of the disease. It is worth noting that changes in medial geniculate body is more related to the severity of disease than that of anterior corona radiata and anterior limb of internal capsule. The more serious the disease is, the more damage of the medial geniculate body. This may be because of that the medial geniculate body is the converging site of multiple inputs from the lower auditory nuclei, which may be more sensitive to injury. Medial geniculate body has also been associated with tinnitus by inhibiting the afferent from the inferior colliculus and thalamic reticular nucleus. Thus, medial geniculate body has been used as the main target in some pharmacological experiments that enhance tonic inhibition in the treatment of auditory diseases (Richardson et al., 2012). Our finding may provide a marker for the treatment of SSNHL, which can be monitored during the treatment. On the other hand, the decrease of QA was only found in the contralateral medial geniculate body but not in the ipsilateral side. Previous studies have reported that the contralateral pathway is more important than the ipsilateral pathway in the processing of auditory stimulation (Khosla et al., 2003; Langers et al., 2005), of which 70–80% from the contralateral side and 20–30% comes from the ipsilateral side. Furthermore, the anterior limb of the internal capsule is believed to be involved in cognitive function, such as decision-making, attention, memory and so on (Nanda et al., 2017). For example, the change of QA value in this area may be related to the psychological problems such as anxiety and depression which often occur in patients with sudden hearing loss (Arslan et al., 2018). Furthermore, Husain et al. (2011) and Boyen et al. (2013) have also found similar changes of the frontal lobe which is associated with memory and higher cognitive processes in patients with hearing impaired. Consistently, the current finding of damage in the anterior limb of the internal capsule may imply the cognitive impairment in this disease.

Some previous studies have also supported the current findings. Huang et al. (2015) have found decreased FA values in regions along the auditory neural pathway using DTI in SSNHL patients, which suggests the presence of a demyelinating process in the underlying microstructures along the auditory pathway. In addition, Husain et al. (2011) also found the changes of thalamic anterior radiation, and corona radiata in patients with hearing loss, which is mainly manifested in the decrease of FA values of nerve fibers in the inner capsule. However, unlike Chang et al. (2004), Lin et al. (2008), and Wu et al. (2009) found in patients with long term sensorineural hearing loss, we did not found changes in the pathway of lateral lemniscus, olivary nucleus or subcortical inferior colliculus. This inconsistency may be due to their object of study was patients with long-term sensorineural hearing loss. Long-term hearing loss or compensatory mechanisms may lead to more extensive fiber damage (axon loss or demyelination). Other fiber expansion to the region may also lead to more disorder of white matter fiber. Consistent with Kim et al. (2009), patients in present study were in the acute stage of this disease, in which it may not occur such extensive injury or compensation in a short time. The current finding indicates that the course of disease may be related to the extent of brain nerve damage. With the prolongation of the course of disease, the range and degree of the injury will continue to progress. It suggests that early treatment may effectively control brain nerve damage and improve the prognosis of patients.

Tinnitus is the main complication of SNNHL. Previous studies have shown that the central auditory system is related to the production and maintenance of tinnitus (Noreña and Eggermont, 2003; Lanting et al., 2008), and tinnitus may also lead to changes in white matter and gray matter (Landgrebe et al., 2009; Benson et al., 2014). When compared to hearing loss patients without tinnitus, Boyen et al. (2013) have found increased gray matter volume in the primary auditory cortex in patients with tinnitus. The primary auditory cortex deals with simple auditory stimuli, such as pure tone and noise (Mirz et al., 1999). The increase of gray matter volume in primary auditory cortex may be related to continuous internal sound stimulation. In addition, Boyen et al. (2014) have further found the functional connection was significantly reduced between the inferior colliculus and the auditory cortex in patients with sensorineural hearing loss with tinnitus compared to patients without tinnitus, which suggests the thalamic dysfunction that affects the transmission of auditory information from hypothalamus to auditory cortex. However, other studies of sensorineural hearing loss have not found difference in functional connection between patients with and without tinnitus (Luan et al., 2019). Compared with normal people, Husain et al. (2011) have also found changes both in gray matter and white matter in the adjacent region of auditory cortex only in hearing loss patients with hearing loss alone and found no changes in patients with hearing loss and tinnitus, which suggests hearing loss has the greatest influence on neural tissue rather than tinnitus. Accordingly, in the present study, it was postulated that the brain area was less likely differentially affected between the two groups due to the short-term stage of patients with tinnitus that may not have a profound effect on brain regions like long-term tinnitus. Meanwhile, a small number of patients without tinnitus which limited the further clarification between the two groups in the present study. The relationship between hearing loss and tinnitus may be complex rather than simple superposition effect, which would be investigated in the further study.

Furthermore, Yeh et al. (2013) have compared FA, GFA, and QA, and found that QA is less sensitive to the partial volume effects of crossing fibers, free water, and non-diffusive materials. QA also has less noise, and the QA assisted traction imaging has better spatial resolution than the FA assisted and GFA assisted traction imaging. In this study, we also found the QA value showed significant difference between the SSNHL group and the control group, but the difference of GFA was not obvious, which supports that QA has advantage in reflecting the changes of white matter fibers. This also suggests that the demyelination of nerve fibers may be the main pathological change, while the nerve fibers are relatively intact.

There are several limitations to our study. First, our patients’ data were obtained from one hospital, which may limit a generalized application of these findings. Secondly, the current study is a cross-sectional study involved in patients at acute stage. A longitudinal study is further required to clarify the white matter alteration along the course of this disease. Thirdly, the effects of concomitant symptom such as tinnitus, dizzy and psychological problems on brain structure and function needs further study.

## Conclusion

In conclusion, the present study demonstrated that the microstructural damages in the auditory neural pathway, especially in the contralateral medial geniculate body that was related to the severity of the disease, exhibited in the acute stage of patients with SSNHL, This study provided imaging evidence for the brain changes and helped us understand the pathophysiological changes of diseases better. The QA value may be a potential biomarker of this disease, which can be used to quantitatively analyze the progress of the disease and medicinal efficacy.

## Data Availability Statement

The datasets for this article are not publicly available because our team are doing further study. Requests to access the datasets should be directed to TJ, 1164003407@qq.com.

## Ethics Statement

The studies involving human participants were reviewed and approved by the Ethics Review Committee of the Beijing Chaoyang Hospital. The patients/participants provided their written informed consent to participate in this study.

## Author Contributions

ZZ and XJ contributed to research and writing. ZZ, XG, YL, and YZ contributed to study design and data collection. ZZ, XJ, and XG completed the reprocessing and data analysis. TJ and JY acted as an independent adjudicator for any discrepancies in this process and provided critical revision of the manuscript.

## Conflict of Interest

The authors declare that the research was conducted in the absence of any commercial or financial relationships that could be construed as a potential conflict of interest.
