# Supplementary material for: White Matter Abnormalities of Auditory Neural Pathway in Sudden Sensorineural Hearing Loss Using Diffusion Spectrum Imaging: Different Findings From Tinnitus
Source: Front Neurosci. 2020 Mar 25;14:200. doi: 10.3389/fnins.2020.00200 (PMC7109467; doi:10.3389/fnins.2020.00200)
Supplement: Supplementary file 1 [file Table_1.DOCX]

| **Supplementary Table. The P value of DSI parameters obtained from the left and right sides comparisons between SSNHL**^†^ **patients with tinnitus or dizzy and those without.** | | | | | | | | |
| --- | --- | --- | --- | --- | --- | --- | --- | --- |
| **Variable** | **With tinnitus or without** | | | | **With dizzy or without** | | | |
|  | **Left-sided SSNHL** | | **Right-sided SSNHL** | | **Left-sided SSNHL** | | **Right-sided SSNHL** | |
|  | QA^‡^ | GFA**^§^** | QA | GFA | QA | GFA | QA | GFA |
| Superior olivary nucleus |  |  |  |  |  |  |  |  |
| Left | 0.165 | 0.467 | 0.641 | 0.980 | 0.955 | 0.749 | 0.885 | 0.256 |
| Right | 0.657 | 0.689 | 0.329 | 0.791 | 0.325 | 0.310 | 0.355 | 0.076 |
| Inferior colliculus |  |  |  |  |  |  |  |  |
| Left | 0948 | 0.655 | 0.653 | 0.877 | 0.554 | 0.998 | 0.825 | 0.323 |
| Right | 0.752 | 0.995 | 0.567 | 0.235 | 0.417 | 0.351 | 0.208 | 0.020 |
| Medial geniculate bodies |  |  |  |  |  |  |  |  |
| Left | 0.910 | 0.764 | 0.198 | 0.619 | 0.186 | 0.033 | 0.558 | 0.286 |
| Right | 0.534 | 0.488 | 0.871 | 0.741 | 0.662 | 0.059 | 0.843 | 0.777 |
| Lateral lemniscus |  |  |  |  |  |  |  |  |
| Left | 0.851 | 0.276 | 0.980 | 0.596 | 0.835 | 0.773 | 0.501 | 0.790 |
| Right | 0.869 | 0.552 | 0.422 | 0.736 | 0.955 | 0.375 | 0.786 | 0.834 |
| Anterior limb of internal capsule |  |  |  |  |  |  |  |  |
| Left | 0.173 | 0.073 | 0.477 | 0.294 | 0.462 | 0.886 | 0.396 | 0.711 |
| Right | 0.521 | 0.362 | 0.464 | 0.673 | 0.123 | 0.344 | 0.634 | 0.390 |
| Posterior limb of internal capsule |  |  |  |  |  |  |  |  |
| Left | 0.828 | 0.720 | 0.329 | 0.888 | 0.343 | 0.915 | 0.940 | 0.691 |
| Right | 0.849 | 0.710 | 0.379 | 0.350 | 0.205 | 0.687 | 0.919 | 0.666 |
| Heschl |  |  |  |  |  |  |  |  |
| Left | 0.797 | 0.573 | 0.704 | 0.270 | 0.235 | 0.433 | 0.237 | 0.573 |
| Right | 0.995 | 0.952 | 0.379 | 0.500 | 0.146 | 0.217 | 0.357 | 0.814 |
| Superior temporal gyrus |  |  |  |  |  |  |  |  |
| Left | 0.463 | 0.177 | 0.680 | 0.472 | 0.523 | 0.599 | 0.863 | 0.308 |
| Right | 0.371 | 0.175 | 0.545 | 0.617 | 0.186 | 0.159 | 0.609 | 0.859 |
| Meddle temporal gyrus |  |  |  |  |  |  |  |  |
| Left | 0.609 | 0.659 | 0.396 | 0.507 | 0.378 | 0.865 | 0.456 | 0.407 |
| Right | 0.379 | 0.505 | 0.584 | 0.588 | 0.206 | 0.189 | 0.775 | 0.905 |
| Inferior temporal gyrus |  |  |  |  |  |  |  |  |
| Left | 0.423 | 0.664 | 0.172 | 0.289 | 0.589 | 0.672 | 0.418 | 0.217 |
| Right | 0.953 | 0.918 | 0.630 | 0.273 | 0.606 | 0.866 | 0.871 | 0.669 |
| Anterior corona radiata |  |  |  |  |  |  |  |  |
| Left | 0.590 | 0.298 | 0.587 | 0.835 | 0.392 | 0.863 | 0.995 | 0.155 |
| Right | 0.781 | 0.617 | 0.660 | 0.942 | 0.515 | 0.785 | 0.842 | 0.003 |
| Posterior corona radiata |  |  |  |  |  |  |  |  |
| Left | 0.659 | 0.404 | 0.615 | 0.250 | 0.232 | 0.844 | 0.986 | 0.705 |
| Right | 0.873 | 0.808 | 0.640 | 0.190 | 0.593 | 0.488 | 0.825 | 0.841 |
| Acoustic radiation |  |  |  |  |  |  |  |  |
| Left | 0.718 | 0.697 | 0.358 | 0.295 | 0.850 | 0.508 | 0.794 | 0.382 |
| Right | 0.861 | 0.984 | 0.976 | 0.119 | 0.555 | 0.979 | 0.933 | 0.160 |
| Brodman 41 area | 0.926 | 0.789 | 0.619 | 0.032 | 0.754 | 0.945 | 0.627 | 0.635 |
| Brodman 42 area | 0.616 | 0.386 | 0.636 | 0.874 | 0.960 | 0.661 | 0.423 | 0.290 |

† SSNHL means sudden sensorineural hearing loss.

‡ QA means quantitative anisotropy.

**§** GFA means generalized fractional anisotropy.

.
